# Caregivers’ Quality of Life and Quality of Services for Children with Cancer: A Review from Iran

**DOI:** 10.5539/gjhs.v5n3p173

**Published:** 2013-03-04

**Authors:** Homeira Sajjadi, Meroe Vameghi, Mehdi Ghazinour, Mohammadreza KhodaeiArdakani

**Affiliations:** 1 Social Determinants of Health Research Center, University of Social Welfare and Rehabilitation Sciences, Tehran, Iran; 2 Department of Social Work, Umeå University, Umeå, Sweden; 3 Psychiatry Department, University of Social Welfare and Rehabilitation Sciences, Tehran, Iran

**Keywords:** quality of life, quality of services, caregivers, children, cancer

## Abstract

**Objective:**

Caregivers of cancer patients are exposed to different types of psychosocial stress which influence their quality of life. The purposes of this study were to assess quality of life in caregivers of children with cancer and to investigate the relationship between the caregivers’ quality of life and quality of services in the social work section.

**Method:**

125 caregivers were recruited. Quality of life was measured by the Iranian version of the WHOQOL-BREF questionnaire and data concerning service quality were obtained from the Iranian version of the SERVQUAL questionnaire.

**Findings:**

Scores in physical health, psychological status and environmental conditions for caregivers of children with cancer were significantly lower than the general population. There was a negative correlation between the tangible domain of SERVQUAL and the psychological status and environmental conditions of quality of life. The tangible dimension was the only aspect of service quality to predict caregivers’ quality of life regarding psychological status and environmental conditions.

**Conclusion:**

Caregivers of children with a disease are care consumers and, like all consumers, they expect good service. Delivering high quality services consistently is difficult but profitable for a service organization. In other words, trying to deliver more appropriate services than patients expect to receive from their social work care is one of the most reliable ways to promote caregivers’ satisfaction and quality of life.

## 1. Introduction

Reforming the successful primary health care programs in Iran resulted in a significant improvement in health status, i.e., decreasing the under-five mortality rate from 73 in 1990 to 32 in 2008 in disadvantaged areas on a national level (UNICEF, 2010). However, based on the report on social determinants of health office (2008), non-communicable diseases are currently the most important cause of mortality and morbidity in Iran. Regarding the same report, non-communicable diseases account for 45% of the total all-cause disease burden for males and 33% for females and, for the majority of children in Iran, communicable diseases are no longer the major cause of death and morbidity. One of the main causes of death among non-communicable diseases is cancer. Cancer accounted for 6.7 million deaths in 2002 and the death toll worldwide is estimated to be 10.3 million by 2020. Each year more than 160,000 children are diagnosed with cancer and it is estimated that 90,000 will eventually die of the disease. Most of them could be cured if essential treatment and care were accessible (WHO and UICC, 2005). In Iran, it was estimated that the incidence rate had increased from nine to 15 per 100,000 children annually in 2008 (Faranoush, 2010).

The process of cancer management with regard to tests, treatment, care arrangements, work, changing roles, financial circumstances and bereavement does not only affect the individual living with cancer, but also the caregivers (Jakobsson, Ekman, & Ahlberg, 2008; Ryan, Howell, Jones, & Hardy, 2008; da Silva et al., 2010; Lin et al., 2009; Alptekin, Gönüllü, Yücel, & Yariş, 2010). It presents numerous recurrent stressors for children and their families (Cappelleri et al., 2008).

Currently, formal and informal caregivers are considered a valuable component pertaining to the treatment of patients (Caqueo-Urízar, Gutiérrez-Maldonado, & Miranda-Castillo, 2009). In all likelihood, this is firstly because of the potential to increase the survival time of children with cancer (Glajchen, Blum, & Calder, 1995; Witt et al., 2010) which has led to the necessity of longer caregiving for them. Secondly, the parents are often the primary reporters of symptoms and the main decision makers on behalf of their child. Thus, considering aspects of the caregivers’ wellbeing and their quality of life must be important when it comes to improving the management of childhood cancer (Barakat, Marmer, & Schwartz, 2010; da Silva, Jacob, & Nascimento, 2010; Kondo-Endo et al., 2009). Quality of life (QOL) has been defined in a variety of ways depending on the context and the field of research (Vellone, Piras, Talucci, & Cohen, 2008). The World Health Organization Quality of Life (WHO QOL (Group define QOL as the individuals’ perception of their position in life in the context of the culture and the value system in which they live and in relation to their goals, expectations, standards and concerns (WHO, 1966). As some studies show, a higher level of caregivers’ QOL seems to improve the outcome of the treatment of the children (Vellone et al., 2008; Kondo-Endo et al., 2009).

According to our search, scarce data are available on the QOL of caregivers of children with cancer. However, the results of some studies on the QOL of caregivers of children with chronic and developmental disease have shown poor QOL of the caregivers (Cappelleri et al., 2008; Caqueo-Urízar et al., 2009; Kondo-Endo et al., 2009; Hatzmann, Maurice-Stam, Heymans, & Grootenhuis, 2009; Yuen Shan Leung & Wai Ping Li-Tsang, 2003).

Poor caregiver QOL would increase health care costs and create economic problems for caregivers (Challis, 1992). A number of studies have illustrated that qualified health care services may ameliorate a number of cancer-related problems and improve well-being and the quick recovery of patients (Parlee, 1999; Glajchen et al., 1995). It is important that primary caregivers (e.g., parents) and secondary care specialists (e.g., social workers) have an understanding of the care needed and the service available. There is also a need for health care providers to understand the patients’ expectations regarding the quality of the social work service to monitor and thus improve service quality (Wisniewski & Donnelly, 1996; Chou, Chen, Woodard, & Yen, 2005; Goldbeck, 2001). MAHAK (the Society to Support Children with Cancer) is the only non-governmental organization in Tehran, the capital city of Iran, which supports children with cancer and their families. The social work section of MAHAK is in constant contact through offices situated in hospitals. The support services include registration, insuring the child and his/her family (if they are not already covered by insurance), paying for medical procedures, and also covering the costs of psychological services, clothing, and educational expenses as well as providing housing for families from far away during treatment.

We were unable to find any Iranian studies on the QOL of caregivers of children with cancer. Because of the dearth of information on this issue in Iran and the importance of the role of social services for the wellbeing of families and children, we decided to assess the QOL of caregivers of children with cancer in relation to gender and educational level and differences with the normal population of Tehran. In addition, we tried to assess the quality of social services in the MAHAK institute, in relation to gender and educational level, as a major social service agent for children with cancer in Iran and its relationship with the QOL of caregivers. We also investigated the relationship between the domains of QOL in caregivers (physical health, psychological status, social relationships and environmental conditions) and the domains of quality of services in the social work section (tangible, reliability, responsiveness, assurance and empathy).

## 2. Materials and Methods

A non-experimental, co-relational research design was used to determine the QOL of caregivers and the relationship to the quality of services in the social work section.

### 2.1 Data Collection

The participants were 125 informal caregivers, mostly the parents of children under 14 receiving treatment or care for their cancer and supported in hospitals covered by MAHAK including Aliasghar hospital, Bahrami hospital, Mophid children’s hospital, Darabad hospital, Shariati hospital and Markazetebi children’s hospital. Data were gathered from June to October 2010. Informed consent was obtained for all participants. Those caregivers who agreed to participate were recruited to the study.

The caregivers answered the questions on their own. Trained investigators assisted the caregivers by reading the questions out to them when needed to alleviate pressure for illiterate caregivers. The time required to fill out the two questionnaires ranged from 30 to 40 minutes.

### 2.2 Instruments

#### 2.2.1 The WHOQOL – BREF Questionnaire

In this study, the validated Iranian version of the WHO QOL questionnaire was used (WHOQOL-BREF) (Usefy et al., 2010). It is a short version of the 100-scale instrument, comprising 26 items, and reflects the multi-dimensional nature of QOL; it also emphasizes subjective experiences rather than objective life conditions and it focuses upon the respondent’s perceived QOL (WHO, 1996). The WHOQOL-BREF was developed in a wide range of cultural and clinical settings (Lin et al., 2009; Alptekin et al., 2010; Rusli, Edimansyah, & Naing, 2008). It contains four domains, namely physical health, psychological status, social relationships and environmental conditions. Physical health is measured by seven items: pain, dependence on medical aids, energy, mobility, sleep and rest, activities of daily living and work capacity. The psychological domain is measured by six items: positive feelings, personal belief, concentration, body image, self-esteem and negative feelings. Social relationships are measured by three items, i.e., personal relationships, social support and sexual life. Environmental conditions comprises eight items focusing on financial resources, security, health and social care, home environment, access to information, physical environment, leisure activities and transportation (WHO, 1996). Items rating QOL overall and subjective satisfaction with health are not included in the domains. An Iranian validation study showed that Cronbach’s alphas for the four domains of the WHOQOL-BREF were satisfactory (physical health = 0.81, psychological status = 0.78, social relationships = 0.82, and environmental conditions = 0.80) (Usefy et al., 2010). The domain score is converted to a transformed score (ranging from 4 to 20) to enable comparison between domains. A higher score denotes a higher QOL. The domain scores were computed on the basis of WHO profiles (WHO, 1996).

#### 2.2.2 The SERVQUAL Questionnaire

The SERVQUAL (service quality) questionnaire is a reliable and valid multiple-item scale (Chou et al., 2005). It is used in a variety of service industries (Babakus & Mangold, 1992; Wisniewski & Donnelly, 1996), and was developed by Parasuraman, Zeithaml and Berry (1998). They defined service quality as the difference between customer expectations and customer perceptions. Measuring QOL from the patient’s perspective with the SERVQUAL instrument has increasingly been used and accepted in health care (Chou et al., 2005; Lin et al., 2009). This tool provides information about patient perceptions of the current service delivery and about their expectations of service managers and associated decision-makers, thus enabling a closer matching of service delivery to expectations and needs and reshaping of the system to focus on the impact these activities have on satisfaction (Wisniewski & Donnelly, 1996). Service quality is measured across five major dimensions: (1) tangible: physical facilities, equipment, and the appearance of personnel (four items, E1–E4)); (2) reliability: the ability to perform the promised service dependably and accurately (five items, E5–E9); (3) responsiveness: willingness to help patients and provide prompt service (four items, E10–E13); (4) assurance: knowledge and courtesy of employees and their ability to inspire trust and confidence (four items, E14–E17); (5) empathy: caring, individualized attention toward patients (five items, E18–E22) (Lam, 1997). In this study, the Iranian version of SERVQUAL was used. An Iranian validation study showed that Cronbach’s alphas for the five domains were satisfactory (tangible = 0.75, reliability = 0.74, responsiveness = 0.72, assurance = 0.75, and empathy = 0.82) (Nobahar, 2007). The questionnaire contains 22 pairs of Likert-type items (1 = strongly disagree and 7 = strongly agree). The first section of this instrument has 22 statements relating to caregivers’ expectations on the quality of the service that an excellent social work section should offer and 22 corresponding items relating to performance perceptions of the quality of service actually delivered. Service quality is measured by the difference in scores (the gap scores) between the expected level and the perceived level of service provided. A positive gap score means that the caregivers’ expectations are more than met.

The demographic variables were caregivers’ gender and educational level. The data from the demographic questionnaire were used to assess statistical differences between the groups regarding the requested information.

### 2.3 Statistical Analysis

To assess the QOL of caregivers of children with cancer, the data and measures used were as follows: we compared the QOL scores of caregivers versus the general population via a t-test (section 3.1); we compared the QOL scores of caregivers’ QOL by gender and education level via a t-test and one-way ANOVA (analysis of variance) (section 3.2). To assess the quality of social work section services in the MAHAK institute in relation to the gender and education level of caregivers, a t-test and one-way ANOVA were used (section 3.3). To investigate the relationship between the caregivers’ QOL and the quality of social work section services provided to them, Pearson’s correlation was used, and to investigate whether the quality of services can predict QOL for each domain, a stepwise linear regression was performed (section 3.4). The software SPSS 17.0 was used for the statistical analyses.

## 3. Results

Out of the 127 questionnaires, two were not included in the study because of missing data. The study group then consisted of 125 caregivers (79 mothers, 41 fathers, 4 sisters, and 1 aunt) of 125 children affected by cancer. In other words, the sample was 67.2% female and 32.8% male. The level of education was as follows: 12.83% illiterate, 26.46% primary school, 28.21% guidance (middle school), 21.38% high school and 11.12 % university students or graduates.

### 3.1 Comparison between the QOL of Caregivers and the General Population

Among caregivers the mean (SD) physical health and psychological status scores were 13.68 (2.8) and 11.76 (3.25), respectively. As for social relationships and environmental conditions, the scores were 13.73 (3.27) and 11.51 (2.85), respectively. In the general population in Tehran, the mean (SD) physical health and psychological status scores were 14.8 (2.3) and 13.7 (2.5), respectively. Regarding social relationships and environment conditions, they were 14.0 (2.4) and 12.6 (2.4), respectively (Nedjat et al., 2007). The caregivers’ QOL scores in all domains were lower than the scores for the general population, and the differences were significant for the domains of physical health (p < 0.001), psychological status (p < 0.000), and environmental conditions (p < 0.001). The greatest difference in scores was for psychological status; the psychological domain had the lowest score.

### 3.2 Comparison of Caregivers’ QOL by Gender and Education Level

Female caregivers scored lower than males in the domains physical health, psychological status and social relationships but no statistically significant differences were observed within them (p > 0.05). Illiterate males had higher scores in the three domains of QOL: physical health, psychological status and environment conditions. Males with higher education had higher scores in social relationships. Illiterate females had higher scores in physical health and psychological status, but the lowest scores in psychological status and social relationships were for females with higher education. No significant differences were found for caregivers’ QOL between educational levels (See Table 1).

**Table 1:** Comparison of caregivers’ QOL by gender and educational level

	Q1 (mean ±SD)	Q2 (mean ±SD)	Physical Health (mean ± SD)	Psychological Status (mean ± SD)	Social Relationships (mean ± SD)	Environmental Conditions (mean ± SD)
**Female**						
Illiterate	2.90±1.04	3.90±0.83	14.93±2.30	11.45±3.94	14.67±2.60	11.42±3.49
Primary school	3.33±0.76	3.83±1.17	13.55±3.72	11.43±3.27	13.72±3.40	11.66±3.14
Guidance	3.05±0.97	3.89±0.87	13.26±2.58	11.66±2.50	12.77±2.86	11.54±3.08
High school	2.64±1.08	3.14±1.35	13.85±2.51	11.19±3.05	14.33±4.22	11.23±2.60
University	2.66±0.87	3.33±0.86	13.40±2.49	10.81±3.72	12.30±3.64	11.50±3.33
**Male**						
Illiterate	2.00±0.82	4.70±0.50	15.30±2.49	14.00±4.45	14.67±5.66	11.87±1.10
Primary school	2.71±0.75	3.57±0.78	13.14±2.42	11.88±3.28	14.48±1.62	11.28±3.22
Guidance	2.71±1.14	3.57±0.65	13.59±3.29	12.19±3.12	14.00±2.75	11.82±2.67
High school	3.09±0.54	4.27±0.79	13.61±1.46	12.54±3.62	14.06±2.76	10.86±2.20
University	3.25±0.50	4.25±0.50	14.57±2.00	13.40±1.64	15.00±2.28	10.87±3.20
**Gender**						
Female	3.02±0.99	3.58±1.10	13.71±3	11.40±3.14	13.59±3.40	11.46±3.09
Male	2.80±0.88	3.95±0.78	13.78±2.50	12.54±3.23	14.27±2.80	11.37±2.51

Q1 = Overall perception of QOL Q2 = Overall perception of health.

### 3.3 Comparison of Quality of Social Work Section Services by Gender and Education Level of Caregivers

The mean of gaps between caregivers’ expectations and perceptions were all greater than zero, as shown in Table 3. The mean (SD) of gaps for the five dimensions were as follows: tangible 1.53 (1.34), reliability 0.78 (1.25), responsiveness 0.87(1.37), assurance 0.76 (1.34), and empathy 1.51 (1.75). Since all mean gap scores were positive, the subjects’ expectations were greater than their perceptions (actual) of the service provided; thus, the caregivers were unsatisfied with the social work section services. Male caregivers were more dissatisfied than females, but the difference was only significant in the responsiveness domain. Males with higher education (university and high school) were more dissatisfied in relation to the dimensions tangible, reliability, responsiveness, assurance and empathy, although the differences in service quality were not significant between different educational levels (See Table 2).

**Table 2:** Comparison of quality of social work section services (gap mean) by gender and educational level of caregivers

	Tangible (Gap mean±SD)	Reliability (Gap mean±SD)	Responsiveness (Gap mean ± SD)	Assurance (Gap mean ± SD)	Empathy (Gap mean ± SD)
**Female**					
Illiterate	1.64±1.46	0.65±0.66	0.70±0.79	0.64±0.76	1.67±2.00
Primary school	1.07±1.25	0.38±1.16	0.56±1.55	0.68±1.54	1.44±1.86
Guidance	1.35±1.20	0.70±1.11	0.71±1.22	0.56±1.12	1.06±1.65
High school	1.93±1.47	1.24±1.51	0.87±1.10	0.91±1.21	1.28±1.60
University	1.26±0.90	0.40±0.88	0.21±1.18	0.96±1.59	1.46±1.56
**Male**					
Illiterate	2.06±1.55	0.20±0.28	1.62±1.48	0.19±0.24	0.60±0.43
Primary school	1.66±1.24	1.20±2.01	1.64±1.79	1.14±1.75	2.80±1.99
Guidance	1.31±1.15	0.71±0.56	0.57±0.67	0.46±0.78	1.64±1.75
High school	1.54±1.72	1.24±1.88	1.50±1.75	0.89±1.87	1.54±1.48
University	2.75±1.51	1.02±0.68	2.37±2.25	2.06±1.90	2.52±2.61
**Female**	1.41±1.32	0.63±1.14	0.60±1.25*	0.69±1.14	1.32±1.68
**Male**	1.66±1.41	0.92±1.34	1.30±1.52	0.83±1.46	1.80±1.77

* p < 0.01 compared to males

**Table 3:** Paired samples t-test of caregivers’ expectations and perceptions of social work section services

Expectations		Perceptions		Statistics	

**E**	**Mean ± SD**	**P**	**Mean ± SD**	**Paired t-test**	**p value**
**E1**	6.06±1.41	**P1**	4.77±1.52	8.15	0.000
**E2**	6.48±0.98	**P2**	5.39±1.34	7.83	0.000
**E3**	6.77±0.44	**P3**	6.06±1.24	6.14	0.000
**E4**	6.42±1.16	**P4**	3.64±2.33	11.40	0.000
**E5**	6.78±0.55	**P5**	5.93±1.40	6.25	0.000
**E6**	6.80±0.59	**P6**	6.12±1.41	4.79	0.000
**E7**	6.77±0.49	**P7**	5.97±1.64	5.34	0.000
**E8**	6.84±0.39	**P8**	5.96±1.52	6.34	0.000
**E9**	6.78±0.57	**P9**	6.16±1.29	4.99	0.000
**E10**	6.60±0.87	**P10**	5.49±1.73	6.18	0.000
**E11**	6.60±0.82	**P11**	5.79±1.47	5.67	0.000
**E12**	6.84±0.43	**P12**	6.01±1.50	5.90	0.000
**E13**	6.28±1.15	**P13**	5.69±1.61	3.59	0.000
**E14**	6.81±0.49	**P14**	6.00±1.46	5.81	0.000
**E15**	6.86±0.45	**P15**	5.93±1.69	5.98	0.000
**E16**	6.90±0.32	**P16**	6.31±1.28	5.19	0.000
**E17**	6.70±0.64	**P17**	6.01±1.40	4.87	0.000
**E18**	6.66±0.69	**P18**	4.99±2.23	8.24	0.000
**E19**	6.81±0.51	**P19**	5.80±1.48	7.54	0.000
**E20**	6.39±1.31	**P20**	4.96±2.10	6.57	0.000
**E21**	6.65±0.69	**P21**	5.04±2.00	8.71	0.000
**E22**	6.59±1.03	**P22**	5.02±2.03	8.16	0.000
**Total**	**6.65±0.36**	**Total**	**5.59±1.13**	**10.13**	**0.000**

In total, 23 paired t-tests (22 on E&P plus 1 on average) were conducted (see Table 3). The results showed that for all dimensions, caregivers had significantly higher scores for expectations than for perceptions. The highest scores for expectations and perceptions were on the items “social workers in the social work section are consistently courteous with the patient (you)” (E16 & P16), and caregivers also had the lowest perceptions concerning materials associated with the social work service (such as pamphlets or posters) (E4).

### 3.4 Correlation between Caregivers’ QOL and the Quality of Social Work Section Services

There was a negative significant correlation between the *tangible* domain of SERVQUAL and *psychological status* (r = -0.217, p < 0.05) and *environmental conditions* (r = -0.279, p < 0.01) of caregivers’ QOL. There was also a significant negative correlation between *empathy* of SERVQUAL and *psychological status* (r = -0.211, p < 0.05) and *environmental conditions* (r = -0.268, p < 0.01) in relation to QOL (see Table 4).

**Table 4:** Correlation between caregivers’ QOL and the quality of social work section services

	Physical Health	Psychological Status	Social Relationships	Environmental Conditions
**Tangible**	-0.032	-0.217*	0.009	-0.279**
**Reliability**	-0.089	-0.120	-0.093	0.006
**Responsiveness**	-0.014	-0.003	-0.031	-0.122
**Assurance**	-0.018	-0.60	0.007	0.012
**Empathy**	-0.053	-0.211*	-0.066	-0.268**

* Correlation is significant at the 0.05 level

** Correlation is significant at the 0.01 level

The results showed that *tangible* was the only dimension of service quality to predict caregivers’ QOL in terms of psychological status (β = -0.529, p < 0.01 and R^2^ = 0.047) and environmental conditions (β = -0.83, p < 0.001 and *R^2^ = 0.108*).

## 4. Discussion

The results of this study indicate that caregivers of children with cancer have low QOL. There was a significant negative association between service quality and two domains of QOL, psychological status and environmental conditions. Even though this study found gaps in all domains of service quality in terms of perception, the scores were higher than those of other health center studies. The only domain to predict QOL was tangible, so hospital managers and policy makers should strive to regulate expectations, especially regarding the physical elements of service quality.

Understanding the impact of childhood cancer on caregivers’ QOL and the factors that affect it is central to care development, treatment processes and making effective interventions to boost survival and QOL for children with cancer (Barakat et al., 2010; Chien et al., 2003). This is, to the best of our knowledge, the first Iranian study on the QOL of caregivers’ of children with cancer and the first to evaluate the social work section services for caregivers.

In this study, the caregivers’ QOL in three domains including physical, psychological status and environmental conditions was significantly lower than the QOL of the general population; this clearly shows how caring for a child with a life threatening disease strongly affects different aspects of caregivers’ QOL, especially in the psychological status domain. This finding is in agreement with studies on the QOL of caregivers of brain tumor patients (Parlee, 1999; Chien et al., 2003) and caregivers of children with disabilities (Lin et al., 2009; Yuen Shan Leung & Wai Ping Li-Tsang, 2003). This is probably due to a combination of stress and socio-economic pressure (Witt et al., 2010), and should be noted by policy makers in order to ameliorate detriments to QOL.

In our study, the QOL of females and males did not differ significantly, although the scores for males were higher. Searching for gender differences regarding QOL or the psychosocial status of parents of children with cancer showed that mothers (Caqueo-Urízar & Gutiérrez-Maldonado, 2006) and female caregivers had a lower QOL than other caregivers (Alptekin et al., 2010; Hatzmann et al., 2009; Webb et al., 1998). However Goldbeck (2001) and McCarthy et al. (2009) found no difference. We also found no significant difference between the QOL of caregivers with different educational levels, although some studies have shown higher QOL in better-educated people (Alshubaili, Awadalla, Ohaeri, & Mabrouk, 2007; Awadalla et al., 2007). One possible explanation is that in difficult situations, e.g., caring for a child with a severe and maybe fatal disease, which cause low QOL, socio-demographic variables such as gender and educational level are not central.

The results of the quality of service in this research revealed that there was no difference either in the subjects’ satisfaction scores between males and females (except in the responsiveness domain), or regarding educational level. These results are the same as those of Hall and Dornan (1990) and Lin et al. (2009) regarding gender. In some studies, people with a lower educational level show better satisfaction (Hall & Dornan, 1990; Barlési et al., 2005).

The average scores in expectations of service quality (6.65±0.36) in this study were higher than the scores of quality of services in patients in some studies, but showed similarities with LASIK surgery (expectations = 6.51/7; perceptions = 6.29/7), and kidney disease patients (expectations = 6.50/7; perceptions = 6.14/7) in Taiwan (Lin et al., 2009). This finding might be due to Iranian caregivers being more prone to receiving qualified services. The perception score (5.59±1.13) was also higher than in some studies (Lam, 1997; Lin, Xirasagar, & Laditka, 2004; Rao, Peters, & Bandeen-Roche, 2006).

There were service quality gaps in all five domains, i.e., the tangible, reliability, responsiveness, assurance, and empathy sections. The largest quality gap in this study was for the tangible and empathy dimensions, but not as marked in the latter. This study suggests that social workers should increase their empathy with caregivers through some methods such as conversation with caregivers—not only with their children. Caregivers need to have opportunities to discuss issues regarding their sick children’s future, be referred to psychologists for consultation, and be provided with useful documents regarding treatment and care. Caregivers need formal and informal support to gain information and psychological and emotional support. Especially for people with poor mental health, the need for social support increases QOL (Hatzmann et al., 2009; Webb et al., 1998; Alshubaili et al., 2007; Rusli et al., 2008). The above have already been shown to be effective ways to increase caregivers’ QOL by hospital managers. Policy makers could focus on how to enhance service quality both in relation to information about current service delivery and caregivers’ expectations, thus enabling a closer matching of service delivery to expectations and needs.

## 5. Conclusion

Caregivers of children with a disease are care consumers and, like all consumers, they expect good service. Delivering high quality services consistently is difficult but profitable for a service organization. In other words, trying to deliver more appropriate services than patients expect to receive from their social work care is one of the most reliable ways to promote caregivers’ satisfaction and QOL.

The results of this study can be discussed in the light of some limitations: for instance, all caregivers were included at the time of study and because all caregivers were supported by MAHAK, their answers to the questions regarding the quality of services may be affected by their situation. In addition, the services provided by MAHAK and represented in this study are not representative of all caregivers providing care and treatment within the organization. Another limitation is that the caregivers’ services were received at different points in time and with different duration.

Longitudinal studies would be of value to assess changes in perceptions and expectations could be tracked over time; such studies may be able to highlight better the potential role of socio-demographic and family risks for QOL outcomes during a child’s cancer treatment. Interventional studies could also be used to examine whether better service quality might be a predictor of QOL for children with cancer and their families. Moreover, it is essential that the multifaceted nature of parent–child relationships be evaluated using qualitative methods during treatment in the context of the family.
